# Knowledge of Cancer Risk Factors, Symptoms, and Barriers to Seeking Medical Help among Omani Adolescents

**DOI:** 10.31557/APJCP.2019.20.12.3655

**Published:** 2019

**Authors:** Mohammed Al-Azri, Waleed Ibrahim Al-Saadi, Abdulaziz Al- Harrasi, Sathiya Murthi Panchatcharam

**Affiliations:** 1 *Department of Family Medicine and Public Health, College of Medicine and Health Sciences, Sultan Qaboos University, *; 2 *Research Department, Oman Medical Specialty Board Muscat, Sultanate of Oman. *

**Keywords:** Cancer, knowledge, barriers, adolescents, Oman

## Abstract

**Objective:**

Raising cancer awareness among adolescents should lead to early diagnosis and improve their survival rate into adulthood. This study aims to identify knowledge of cancer risk factors, symptoms and barriers to seek medical help among Omani adolescents.

**Methods:**

A cross-sectional study with Omani adolescents (aged 15-17 years) has been conducted in six schools in Muscat, the capital of Oman. The general Cancer Awareness Measure questionnaire was used to collect the data.

**Results:**

A total of 481 adolescents participated. The average recognition of cancer risk factors and symptoms was low (36.8% and 39.6%, respectively). Cancer risk factors and/or symptoms significantly recognised more in girls compared to boys (χ^2^ = 10.136; Odds ratio [OR] = 2.13 ; 95% confidence interval [CI]= 0.33-3.41; P = 0.001); older (age 17 year) versus younger (aged 15 and 16 years) (χ^2^ = 6.075; OR = 11.68; 95% CI: 1.11-2.53; P = 0.014); those with existing co-morbidities compared to those without (χ^2^ = 4.955; OR = 0.41; 95% CI: 0.18-0.92; P = 0.026); and those who knew someone with cancer compared to those who did not (χ^2^ = 15.285; OR 2.70; 95% CI: 1.62-4.49; P <0.001). The majority of adolescents (88.8%) would seek medical help within the first two weeks of noting cancer symptoms. The most notable barriers to seek medical help were “emotional”. Girls were experienced “emotional barriers” significantly more than the boys (χ^2^ = 11.617; OR = 1.89; 95% CI: 1.31-2.72; P = <0.001).

**Conclusion:**

Adolescents in Oman showed poor cancer awareness with several “emotional” barriers. There is a need to establish and integrate effective cancer educational programs in school curriculums to raise the cancer awareness, address emotional barriers and encourage seeking early medical help. The program could potentially have a life-long impact on encouraging early cancer diagnosis and improving the cancer survival rate.

## Introduction

Cancer is predicted to rank as the leading cause of death and the most important barrier to increasing life expectancy in every country globally in the 21^st^ century (Bray et al., 2018). The burden of cancer has been on the rise over the past few years, with a worldwide estimated incidence of 18.1 million and mortality rate of 9.6 million in 2018 (World Health Organization, 2018). Although the incidence of cancer has been increasing in both developed and developing countries, the majority of deaths occur in developing countries, probably due to a combination of delayed diagnosis and limited access to the latest treatments (Jassem et al., 2013; World Health Organization, 2018). Furthermore, the burden of cancer in developing countries is expected to double over the next two decades as a result of socioeconomic changes, such as increased tobacco use, aging populations and unhealthy lifestyles (Torre et al., 2015). 

The incidence of breast cancer has reportedly increased by 3%-4% annually, and infection-associated cancers, including cervical, stomach, and liver, account for 20% of all cases of cancer in developing countries (Babu, 2008; Bray et al., 2018; de Martel et al., 2012). The prevalence of adulthood obesity in the Gulf Countries has increased at an alarming rate along with industrial development over the last two decades, this is due to significant growth in incomes, rapid urbanization and improved living conditions. Increased rates of obesity, including childhood obesity, overconsumption of high-calorie and low nutrition meals, smoking and air pollution, are all associated with higher incidences of cancer in the Gulf Countries (Alnohair, 2014).

Cancer has been also identified as the leading cause of non-accidental death among teenagers and young people (aged 10–19 years) in developed countries, with approximately 2630 new patients being diagnosed with cancer in the United Kingdom in 2013-2015 (Cancer Research UK, 2019). There has been an increased incidence in the number of cancer cases among teenagers and young people by more than 28% since 1990 (Cancer Research UK, 2019). Although exposure to avoidable cancer risk factors have less of an impact on younger people than adults due to lower rates of exposure over time, awareness of these factors among adolescents are associated with certain protective health-related behaviors, which can build a foundation for healthy adulthood (Kyle et al., 2013). Risk-taking behavior in adolescence have been found to be effective interventional measures in influencing health behaviors in adulthood (Due et al., 2011).

Poor knowledge of cancer signs and symptoms, combined with negative beliefs and attitudes, have been considered to be the main reason for delayed presentation and diagnosis of cancer, particularly if the cancer symptoms are atypical (Jassem et al., 2013). It has been found that certain types of cancer can be cured and treated adequately if detected early and low recognition levels of cancer warning signs have been linked to delays in seeking medical help (Quaife et al., 2014). Despite this, almost half of the reported cancer patients in the Gulf Countries had either regional or distant metastasis at the time of diagnosis, and only 22.6% of patients presented with localized tumors (Nasser Al Hamdan, 2006). 

Teenagers and young adults with cancer have been noted to seek medical help more before a cancer diagnosis (Dommett et al., 2013). However, there several emotional barriers preventing adolescents from seeking early medical help (Hubbard et al., 2014) Raising awareness of cancer symptoms and addressing the barriers to seeking medical help have been found to be effective in promoting early cancer diagnosis (Walter et al., 2012). 

Oman is one of the Gulf Countries located in the south-eastern quarter of the Arabian Peninsula, with an estimated population of 4.6 million, of which 2.5 million are Omani and 2.1 million are expatriates (National Center for Statistics and Information, 2017). Approximately 35% of Omanis are aged below 15 years and only 3.5% are above 65 years old (median age: 22 years), with 21% of the population living in the capital city, Muscat, the most populated city in Oman (National Center for Statistics and Information, 2017). 

A report from the Ministry of Health (MOH) indicated that 75% of the disease burden in Oman is attributable to non-communicable diseases, including cancer, which has increased to a level similar to that in developed countries (Al-Lawati et al.,2008). The incidence of cancer is currently 103.8 per 100,000 of the total population, with 1800 cases reported in 2015. From this, 1,615 (90.0%) were Omani and 185 (10.0%) were expatriates (Ministry of Health, 2015). Breast cancer was the most common cancer among women, while prostate cancer was the most common among men (Ministry of Health, 2015). As in other developing countries, the incidence of cancer is expected to increase and become the leading cause of death over the next 25 years, due to urbanization, an increasing elderly population and a “westernization” of lifestyles (Bray et al., 2018). 

Even though the latest treatments are available, the majority of Omani cancer patients tend to present at advanced stages, at a younger age, and with lower survival rates (Kumar et al., 2011). Furthermore, there are currently no screening programs available for cancer, except for breast cancer, as a program was introduced in 2010 (Ministry of Health, 2010).

Previous studies among the adult Omani population have shown that the majority of the public were not aware of the risk factors and symptoms for most types of cancer (Al-Azri et al., 2017; Al-Azri et al., 2016; Al-Azri et al., 2014). There are also several notable social and cultural barriers contributing to the delays in cancer diagnosis, particularly among vulnerable groups (Al-Azri et al., 2016). To the best of our knowledge, there have been no previous studies conducted among the adolescents in Oman to identify their awareness levels of cancer risk factors, the warning symptoms, anticipated delay time and perceived barriers to seeking medical help. 

## Materials and Methods

*Tool Used to Measure Cancer Awareness*

The Cancer Awareness Measure (CAM) questionnaire is a validated standardized measurement for cancer awareness in the general population (Stubbings et al., 2009). The questionnaire includes three different sections: the first includes 12 close-ended questions with five options, corresponding to the level of agreement with a known risk of developing different common types of cancer; the second includes a questionnaire of 10 possible warning symptoms of cancer that were reported either in the European Code against Cancer or by major cancer organizations; the last section includes a questionnaire which measures the anticipated time before seeking medical help, and perceived barriers to presentation for the warning symptoms of cancer. 

The barriers to seek medical help were further categorized into emotional, practical, and services. The internal reliability and test–retest reliability of CAM questionnaire was found to be high (Stubbings et al., 2009). The CAM questionnaire was translated from English to Arabic and back-translated into English again by several people proficient in both languages, and the Arabic version of the CAM questionnaire has been used in previous studies conducted among adults in Oman (Al-Azri et al., 2014; Al-Azri et al.. 2015; Al-Azri et al., 2016). 

Prior to data collection, a pilot study was conducted to check the clarity of the Arabic version of the CAM questionnaire. A group of 30 adolescent students were asked to reflect upon the meaning of their responses and whether their response had been shaped by their interpretation of individual questions. We recorded the students’ suggested changes to the instrument and their reasons for these changes. However, no changes were suggested by the students. The data from the pilot study were used to test the reliability of the Arabic version of the CAM questionnaire for the current study. Based on the standardized items, the Cronbach’s alpha (α) of the Arabic version of CAM questionnaire in this study was 0.82. 

*Recruitment of schools*

Approximately 21% of total population of Oman reside in Muscat. The majority of the population of Muscat come from all regions Oman. Al-Seeb is a large coastal province in Muscat, located in the northwest, with a total population of 237,816, comprising of 9.5% of the total population of Oman according to 2017 census (National Center for Statistics and Information, 2017). Al-Seeb was selected as it is close to the Sultan Qaboos University (SQU), and was also predicted to provide a good representative sample of adolescents. 

The Ministry of Education in Oman is in charge of managing school education among all stages (Grades 1-12), and the Directorates of General Education are in charge of the implementation of the Ministry’s education plans. Muscat has the largest percentage of government schools, accounting for 23% of all schools in Oman. Approximately 65.5% of schools in Oman are government schools, while 34.5% are private (National Center for Statistics and Information, 2018). 

A total of six government schools, with students in Grades 10-12 (aged 15-17 years) were randomly selected from the Al-Seeb area (three male and three female schools). The Directorates of General Education in Al-Seeb was contacted by letter explaining the purpose of the study and asking their permission to enroll the selected schools in the study. The principals of the selected school were then contacted by the Directorates of General Education. The composition of the selected schools was comparable with others, exhibiting a similar geographical spread and socio-demographic profiles among the students.

*Sample Size Calculation*

To the best of our knowledge, no previous studies have been conducted in Oman among adolescents to determine their awareness levels of cancer risk factors, the warning signs or symptoms, anticipated delays and the perceived barriers to seeking medical help. Therefore, for the sample size calculation, we assumed that Omani adolescents had a 50% awareness level of cancer risk factors and warning symptoms. Thus, with a precision rate of 5% and desired confidence interval (CI) of 95%, we determined that around 400 participants were needed for this study. However, to overcome missing and poor responses, the number of invitees was increased by 20% to 480.

*Recruitment of participants*

This cross-sectional study was carried out between 1st January 2018 to 31st March 2018. Two medical students were trained to use the CAM questionnaire in order to distribute and collect the data from the study’s participants. To meet the aims of the study, data were collected from Omani adolescents aged between 15 and 17 years old. Parents/guardians of the students in Grades 10-12 from each of the selected schools were sent letters explaining the study, as well as consent form to sign on behalf of the students to allow them to participate. The parents/guardians were asked to return the consent forms via the students. 

A time was allocated in the class rooms to meet the students and provide them with verbal and written information about the study. They were also asked to give written consent if they agreed to participate. The CAM questionnaire (Arabic Version) was given to the students to complete. 

*Statistical Methods*

Data were analysed using the Statistical Package for the Social Sciences (SPSS), version 22 (IBM Corp, Chicago, Illinois). For descriptive purposes, categorized variables were presented as numbers and percentages. The Chi-squared test (χ^2^) was used to examine relationships between the CAM variables and dichotomised demographic variables, such as gender (male/female), age (younger [15-16 years] and older [17 years]), presence of co-morbidities (yes/no), and knowing someone with cancer. A multinomial regression model was used to adjust it for the factors; a P value of <0.05 was considered to be statistically significant. 

## Results

*Sample*

All of the invited adolescents agreed to participate in the study (N = 481). There were 258 (53.6%) male and 223 (46.4%) female participants. The ages of the students were 15 (5.0%), 16 (36.8%) and 17 (58.2%) years. Around 7.4% of the students declared that they were obese; 3.4% smoked or had smoked; 0.8% drank alcohol or they had drunk alcohol and 16.8% had a family history of cancer, the majority (48.1%) of which had a second degree relative who had been diagnosed with cancer (Table 1).

*General knowledge of cancer and cancer screening in Oman*

The majority of the adolescents (40.3%) did not know if cancer was a prevalent condition in Oman, however, they did think that cancer could be cured if detected early (84.4%). A change of lifestyle was the most commonly reported contributing risk factor to developing cancer (63.2%), followed by genetics (18.9%), environmental factors (12.9%), purely by chance (3.7%) and gaining weight (1.2%). More than half of the adolescents did not know breast cancer (50.0%), cervical cancer (72.6%) or bowel cancer (71.7%) screening programs in Oman. The majority (61.1%) said they would like to undergo relevant cancer screening tests in the future (Table 2).

*Recognition of cancer risk factors*

**Figure 1:**
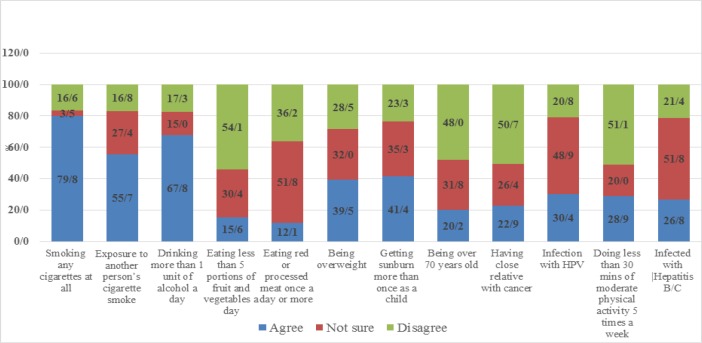
Distribution of Responses to Cancer Risk Factors among the Participants (N = 481).

The average recognition level of all cancer risk factors in the CAM questionnaire was (36.8%); “smoking of cigarettes” was the most commonly recognized cancer risk factors (79.8%), followed by “drinking alcohol” (67.8%), “exposure to another person’s cigarette smoke” (55.7%), “getting sunburnt more than once as a child” (41.4%), “being overweight” (39.5%), “infection with HPV” (30.4%), “doing less than 30 minutes of moderate physical activity 5 times a week” (28.9%), “infection with Hepatitis B or C” (26.8%), “having close relatives with cancer” (22.9%), “being over 70 years old” (20.2%), “eating less than 5 portions of fruit and vegetables a day” (15.6%) and “eating red or processed meat once a day or more” (12.1%) (Figure 1).

The multinomial regression analyses, after being adjusting for other variables, showed that a significantly higher percentage of female adolescents reported higher awareness levels of the following as risk factors of cancer: “smoking cigarettes” (χ^2^ = 10.136; Odds ratio [OR] = 2.13; 95% CI = 0.33-3.41; P = 0.001); “getting sunburnt more than once as a child” (χ^2^ = 4.751; OR = 1.50; 95% CI: 1.04-2.16; P = 0.029); “having a close relative with cancer” (χ2 = 4.742; OR = 1.61; 95% CI: 1.05-2.46; P = 0.029); and “infection with HPV” (χ2 = 8.100; OR = 1.76; 95% CI: 1.19-2.61; P = 0.004) (Table 3).

A significantly higher percentage of adolescents who were 17 years of age (compared to those who were 15 or 16 years) were more aware of the following as cancer risk factors: “doing less than 30 minutes of moderate physical activity 5 times a week” (χ^2^ = 6.075; OR = 11.68; 95% CI: 1.11-2.53; P = 0.014) and “eating less than 5 portions of fruit and vegetables a day” (χ^2^ = 15.283; OR = 3.09; 95% CI: 1.72-5.55; P <0.001). However, a significant number of adolescents who were 17-years-oldcompared to those aged 15 or 16 years were less aware on the following cancer risk factors: “smoking cigarettes” (χ^2^ = 12.810; OR = 0.41; 95% CI: 0.25-0.67; P <0.001) and “drinking alcohol” (χ^2^ = 7.421; OR = 0.58; 95% CI: 0.39-0.86; P = 0.006) (Table 3).

A significantly higher percentage of adolescents who reported to have existing co-morbidities were less aware that “eating less than 5 portions of fruit and vegetables a day” is a risk factor of cancer compared to those without (χ^2^ = 4.955; OR = 0.41; 95% CI: 0.18-0.92; P = 0.026) (Table 3).

A significantly higher percentage of adolescents who reported knowing someone with cancer, compared to those who did not, were more aware of the following as cancer risk factors: “being overweight” (BMI over 25) (χ^2^ = 3.980; OR = 1.63; 95% CI: 1.01-2.63; P = 0.046) and “having a close relative with cancer” (χ^2^ = 15.285; OR = 2.70; 95% CI: 1.62-4.49; P <0.001) (Table 3). 

*Recognition of cancer warning signs or symptoms*

**Figure 2:**
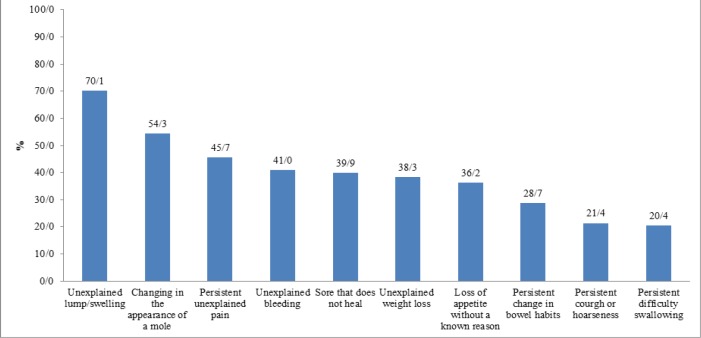
Distribution of Recognition of Signs and Symptoms of Cancer among the Participants (N = 481).

The average recognition level of all cancer symptoms in the CAM questionnaire was 39.6%, with “unexplained lump or swelling” being the most commonly recognised cancer symptom (70.1%), followed by “change in mole appearance” (54.3%), “persistent unexplained pain” (45.0%), “unexplained bleeding” (41.0%), “a sore that does not heal” (39.9%), “unexplained weight loss” (38.3%), “loss of appetite without knowing the reason” (36.2%), “persistent change in bowel/bladder habits” (28.7%), “persistent cough or hoarseness” (21.4%) and “persistent difficulty in swallowing” (20.4%) (Figure 2).

In the multinomial regression analyses, after adjusting for other variables, a significantly higher percentage of female adolescents compared to male reported recognising the following signs or symptoms of cancer: “unexplained lump or swelling” (χ^2^ = 28.544; OR = 3.09; 95% CI: 2.02-4.72; P <0.001), “unexplained persistent pain” (χ^2^ = 4.079; OR =1.45; 95% CI: 1.01-2.08; P = 0.043) and “change in mole appearance” (χ^2^ = 4.847; OR = 1.50; 95% CI: 1.04-2.16; P = 0.028) (Table 4).

A significantly higher percentage of the older adolescents (aged 17 years) compared to the younger group (aged 15 or 16 years) were less likely to recognize “unexplained bleeding” (χ^2^ = 5.680; OR = 0.64; 95% CI: 0.44-0.92; P = 0.017) as a symptoms of cancer (Table 4). 

*The anticipated time to consult a doctor in relation to the recognized cancer symptoms*

The majority of adolescents (88.8%) indicated that they would seek medical help for a symptom they thought might be due to cancer within the first 2 weeks. “Unexplained bleeding” was the most symptoms which would cause the adolescents to seek early medical help (within the first two weeks) (85.0%), followed by “unexplained lump or swelling” (80.7%), “persistent unexplained pain” (75.5%), “a sore that does not heal” (71.7%), “persistent difficulty in swallowing” (68.6%), “persistent change in bowel habits” (65.7%), “change in mole appearance” (60.7%), “persistent cough or hoarseness” (58.4%), “loss of appetite without a known reason” (38.0%) and “unexplained weight loss” (25.4%) (Table 5).

*Barriers to seek medical help*

**Figure 3:**
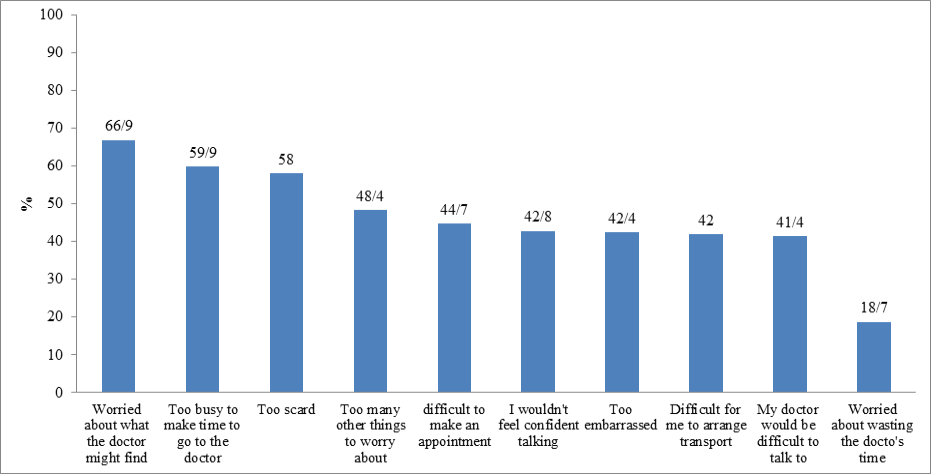
Barriers to Seeking Help among the Participants (N = 481).

The most common emotional barriers to seeking medical help was a “worry about what the doctor might find” (66.9%). The other emotional barriers were “too scared” (58.0%), “would not feel confident talking with a doctor” (42.8%) and “too embarrassed” (42.4%) (Figure 3). 

The most common practice barriers were “too busy to make time to go to the doctor” (59.9%), “too many other things to worry about” (48.4%) and “difficult to arrange transport” (42.0%). Reported service barriers were “difficult to make an appointment with doctor” (44.7%), “my doctor was difficult to talk to” (41.4%) and “worry about wasting doctor’s time” (18.7%) (Figure 3).

In the multinomial regression analyses, after adjusting for other variables, a significantly higher percentage of female compared to male adolescents identified the following as barriers to seeking medical help: “too embarrassed” (χ^2^ = 11.617; OR = 1.89; 95% CI: 1.31-2.72; P <0.001), “too scared” (χ^2^ = 41.208; OR = 3.46; 95% CI: 2.35-5.08; P <0.001) and “worried about what the doctor might find” (χ^2^ = 7.107; OR = 1.69; 95% CI: 1.15-2.49; P = 0.008). However, more males reported “worried about wasting the doctor’s time” (χ^2^ = 5.199; OR = 0.58; 95% CI: 0.36-0.93; P = 0.023) as a barrier compared to females (Table 6).

A significantly higher percentage of older adolescents (aged 17 years) compared to younger group (aged 15 or 16 years) were more likely to identify “difficult to arrange transport to the doctor’s clinic” (χ^2^ = 4.569; OR = 1.50; 95% CI: 01.03-2.17; P = 0.033) as a barrier to seeking medical help (Table 6). 

A significantly higher percentage of adolescents who reported to know someone with cancer (compared to those who did not) were less likely to identify “too busy to make time to go to the doctor” as a barrier to seeking medical help (χ^2^ = 3.943; OR = 0.63; 95% CI: 0.39-1.00; P = 0.047) (Table 6). 

**Table 1:** Socio- Demographic Characteristics of the Participants (N = 481)

Variables	n	%
Gender		
Male	258	53.6
Female	223	46.4
Age in years		
15	24	5.0
16	177	36.8
17	280	58.2
Medical problems		
Diabetes	4	0.8
Hypertension	4	0.8
Obesity	34	7.4
Cancer	2	0.4
Others	45	9.4
Smoking status		
Yes	8	1.7
No	465	96.7
Was but stopped	8	1.7
Drinking alcohol		
Yes	2	0.4
No	477	99.2
Was but stopped	2	0.4
Family history of cancer		
Yes	81	16.8
No	400	83.2
If yes, what type of cancer?		
Lung	8	9.9
Breast	23	28.4
Prostate	2	2.5
Gastric	11	13.6
Liver	1	1.2
Lymphoma	1	1.2
Hematological	9	11.1
Thyroid	2	2.5
Cervical	6	7.4
Others	18	22.2
Specify the relationship to known cancer patient		
First degree	11	13.6
Second degree	39	48.1
Third degree	13	16.0
Others	18	22.2

**Table 2:** General Knowledge of Cancer and Cancer Screening in Oman (N = 481)

Variables	n	%
How common is cancer in Oman?		
Very common	127	26.4
Not common	160	33.3
Don’t know	194	40.3
Do you think that cancer can be cured if detected earlier?		
Yes	406	84.4
No	21	4.4
Don’t know	54	11.2
The most common factor to contribute to the causation of cancer is?		
Lifestyle	304	63.2
Chance	18	3.7
Aging	6	1.2
Environmental factors	62	12.9
Genetic inheritance	91	18.9
Is there a breast cancer screening program in Oman?		
Yes	196	40.7
No	40	8.3
Don’t know	245	50.9
Is there a cervical cancer screening program in Oman?		
Yes	84	17.5
No	48	10.0
Don’t know	349	72.6
Is there a bowel cancer screening program in Oman?		
Yes	92	19.1
No	44	9.1
Don’t know	345	71.7
Would you like to undergo cancer screening test in the future?		
Yes	294	61.1
No	71	14.8
Don’t know	116	24.1

**Table 3:** Recognition of Cancer Risk Factors among the Participants (N = 481)

Variables		Smoking cigarettes		Exposure to another person’s cigarette smoke		Drinking alcohol		Eating less than 5 portions of fruit and vegetables a day		Eating red or processed meat once a day or more		Being overweight (BMI over 25)	
		%	OR	%	OR	%	OR	%	OR	%	OR	%	OR
			(95% CI)		(95% CI)		(95% CI)		(95% CI)		(95% CI)		(95% CI)
Gender	Male (n=258)	74.4	1.00	56.2	1.00	64.3	1.00	18.6	1.00	14.7	1.00	37.2	1.00
	Female (n=223)	86.1	2.13 (1.33-3.41)	55.2	0.96 (0.67-1.37)	71.7	1.41 (0.96-2.07)	12.1	0.60 (0.36-1.00)	9.0	0.57 (0.32-1.01)	42.2	1.23 (0.85-1.77)
Age	15-16 (n=201)	87.6	1.00	60.2	1.00	74.6	1.00	8.0	1.00	10.4	1.00	41.8	1.00
	17 (n=280)	74.3	0.41 (0.25-0.67)	52.5	0.73 (0.51-1.05)	62.9	0.58 (0.39-0.86)	21.1	3.09 (1.72-5.55)	13.2	1.31 (0.74-2.31)	37.9	0.85 (0.59-1.23)
Co-Morbidities	No (n=392)	79.1	1.00	56.1	1.00	67.6	1.00	17.3	1.00	12.5	1.00	40.1	1.00
	Yes (n=89)	83.1	1.30 (0.71-2.39)	53.9	0.92 (0.58-1.45)	68.5	1.04 (0.64-1.71)	7.9	0.41 (0.18-0.92)	10.1	0.79 (0.37-1.67)	37.1	0.88 (0.55-1.42)
Knew someone with cancer	No (n=400)	78.3	1.00	55.3	1.00	66.5	1.00	16.8	1.00	12.5	1.00	37.5	1.00
	Yes (n=81)	87.7	1.97 (0.98-3.99)	58.0	1.12 (0.69-1.82)	74.1	1.44 (0.84-2.47)	9.9	0.54 (0.25-1.18)	9.9	0.77 (0.35-1.69)	49.4	1.63 (1.01-2.63)
Variables		Getting sunburn more than once as a child		Being over 70 years old		Having a close relative with cancer		60-Infection with HPV (Human Papillomavirus)		Doing less than 30 minutes of moderate physical a ctivity 5 times a week		infected with Hepatitis B/C	
		%	OR	%	OR	%	OR	%	OR	%	OR	%	OR
			(95% CI)		(95% CI)		(95% CI)		(95% CI)		(95% CI)		(95% CI)
Gender	Male (n=258)	36.8	1.00	22.1	1.00	19.0	1.00	24.8	1.00	31.0	1.00	23.6	1.00
	Female (n=223)	46.6	1.50 (1.04-2.16)	17.9	0.77 (0.49-1.21)	27.4	1.61 (1.05-2.46)	36.8	1.76 (1.19-2.61)	26.5	0.80 (0.54-1.19)	30.5	2.46 (0.30-1.42)
Age	15-16 (n=201)	44.3	1.00	21.4	1.00	26.9	1.00	32.3	1.00	22.9	1.00	30.3	1.00
	17 (n=280)	39.3	0.81 (0.56-1.18)	19.3	0.88 (0.56-1.38)	20.0	0.68 (0.44-1.04)	28.9	0.85 (0.58-1.26)	33.2	1.68 (1.11-2.53)	24.3	1.04 (0.24-0.74)
Co-Morbidities	No (n=392)	40.3	1.00	21.4	1.00	24.0	1.00	30.9	1.00	29.8	1.00	27.3	1.00
	Yes (n=89)	46.1	1.27 (0.80-2.01)	14.6	0.63 (0.33-1.18)	18.0	0.69 (0.39-1.25)	28.1	0.87 (0.53-1.46)	24.7	0.77 (0.46-1.31)	24.7	1.25 (0.25-0.87)
Knew someone with cancer	No (n=400)	40.0	1.00	19.3	1.00	19.5	1.00	30.5	1.00	28.0	1.00	26.5	1.00
	Yes (n=81)	48.1	1.39 (0.86-2.25)	24.7	1.38 (0.78-2.41)	39.5	2.70 (1.62-4.49)	29.6	0.96 (0.57-1.62)	33.3	1.29 (0.77-2.14)	28.4	4.49 (0.28-1.10)

**Table 4:** Recognition of Cancer Signs and Symptoms among the Participants (N = 481).

Variables		Unexplained Lump/Swelling		Unexplained Persistent Pain		Unexplained Bleeding		Persistent Cough/Hoarseness		Difficulty in Swallowing	
		%	OR	%	OR	%	OR	%	OR	%	OR
			(95% CI)		(95% CI)		(95% CI)		(95% CI)		(95% CI)
Gender	Male (n=258)	59.7	1.00	41.5	1.00	37.6	1.00	19.4	1.00	17.4	1.00
	Female (n=223)	82.1	3.09 (2.02-4.72)	50.7	1.45 (1.01-2.08)	44.8	1.35 (0.94-1.94)	23.8	1.30 (0.84-2.01)	23.8	1.48 (0.95-2.30)
Age	15-16 (n=201)	74.6	1.00	49.8	1.00	47.3	1.00	17.4	1.00	17.9	1.00
	17 (n=280)	66.8	0.68 (0.46-1.02)	42.9	0.76 (0.53-1.09)	36.4	0.64 (0.44-0.92)	24.3	1.52 (0.96-2.40)	22.1	1.30 (0.82-2.06)
Co-Morbidities	No (n=392)	68.6	1.00	45.4	1.00	39.8	1.00	21.4	1.00	20.2	1.00
	Yes (n=89)	76.4	1.48 (0.87-2.53)	47.2	1.07 (0.68-1.70)	46.1	1.29 (0.81-2.05)	21.3	1.00 (0.57-1.74)	21.3	1.08 (0.61-1.89)
Knew someone with cancer	No (n=400)	69.8	1.00	43.8	1.00	40.3	1.00	21.0	1.00	21.0	1.00
	Yes (n=81)	71.6	1.09 (0.65-1.85)	55.6	1.61 (0.99-2.60)	44.4	1.19 (0.73-1.92)	23.5	1.15 (0.65-2.03)	17.3	0.79 (0.42-1.47)
Variables		Change in Appearance of Mole		Sore that Does Not Heal		Unexplained Weight Loss		Loss of Appetite		Change in Bowel Habit	
		%	OR	%	OR	%	OR	%	OR	%	OR
			(95% CI)		(95% CI)		(95% CI)		(95% CI)		(95% CI)
Gender	Male (n=258)	49.6	1.00	37.2	1.00	34.9	1.00	34.1	1.00	26.4	1.00
	Female (n=223)	59.6	1.50 (1.04-2.16)	43.0	1.28 (0.88-1.84)	42.2	1.36 (0.94-1.97)	38.6	1.21 (0.84-1.76)	31.4	1.76 (0.31-1.28)
Age	15-16 (n=201)	55.7	1.00	43.3	1.00	43.3	1.00	37.8	1.00	26.9	1.00
	17 (n=280)	53.2	0.90 (0.63-1.30)	37.5	0.79 (0.54-1.14)	34.6	0.69 (0.48-1.01)	35.0	0.89 (0.61-1.29)	30.0	1.29 (0.30-1.17)
Co-Morbidities	No (n=392)	53.3	1.00	40.6	1.00	39.8	1.00	37.8	1.00	28.3	1.00
	Yes (n=89)	58.4	1.23 (0.77-1.96)	37.1	0.86 (0.54-1.39)	31.5	0.69 (0.42-1.13)	29.2	0.68 (0.41-1.12)	30.3	1.12 (0.30-1.10)
Knew someone with cancer	No (n=400)	53.5	1.00	39.5	1.00	36.8	1.00	34.5	1.00	28.8	1.00
	Yes (n=81)	58.0	1.20 (0.74-1.95)	42.0	1.11 (0.68-1.80)	45.7	1.45 (0.89-2.34)	44.4	1.52 (0.94-2.47)	28.4	2.47 (0.28-0.98)

**Table 5:** The Expected Time to Consult a Doctor in Relation to Recognizing a Possible Cancer Symptom among the Participants (N = 481).

Variables	Within 2 weeks	Within 1 month	Within 6 months	After 6 months	I don’t go
	n (%)	n (%)	n (%)	n (%)	n (%)
Symptom that you thought might be a sign of cancer	427 (88.8)	31 (6.4)	6 (1.2)	3 (0.6)	14 (2.9)
Unexplained bleeding	409 (85.0)	21 (4.4)	13 (2.7)	3 (0.6)	35 (7.3)
Unexplained lump/swelling	388 (80.7)	41 (8.5)	10 (2.1)	3 (0.6)	39 (8.1)
Persistent unexplained pain	363 (75.5)	48 (10.0)	22 (4.6)	4 (0.8)	44 (9.1)
A sore that does not heal	345 (71.7)	61 (12.7)	28 (5.8)	10 (2.1)	37 (7.7)
Persistent difficulty swallowing	330 (68.6)	69 (14.3)	30 (6.2)	6 (1.2)	46 (9.6)
Persistent change in bowel habits	316 (65.7)	55 (11.4)	23 (4.8)	17 (3.5)	70 (14.6)
Changing in the appearance of a mole	292 (60.7)	68 (14.1)	39 (8.1)	8 (1.7)	74 (15.4)
Persistent cough or hoarseness	281 (58.4)	104 (21.6)	28 (5.8)	7 (1.5)	61 (12.7)
Loss of appetite without a known reason	183 (38.0)	100 (20.8)	52 (10.8)	31 (6.4)	115 (23.9)
Unexplained weight loss	122 (25.4)	116 (24.1)	70 (14.6)	28 (5.8)	148 (30.1)

**Table 6:** Participants’ Perception of Barriers to Presentation (N = 481)

Variables		Too embarrassed		Too scared		Worried about wasting the doctor’s time		My doctor would be difficult to talk to		Difficult to make an appointment with my doctor	
		%	OR	%	OR	%	OR	%	OR	%	OR
			(95% CI)		(95% CI)		(95% CI)		(95% CI)		(95% CI)
Gender	Male (n=258)	35.3	1.00	44.6	1.00	22.5	1.00	40.3	1.00	45.7	1.00
	Female (n=223)	50.7	1.89 (1.31-2.72)	73.5	3.46 (2.35-5.08)	14.3	0.58 (0.36-0.93)	42.6	1.10 (0.76-1.58)	43.5	0.91 (0.64-1.31)
Age	15-16 (n=201)	37.3	1.00	57.7	1.00	18.4	1.00	39.8	1.00	49.3	1.00
	17 (n=280)	46.1	1.44 (0.99-2.08)	58.2	1.02 (0.71-1.47)	18.9	1.03 (0.65-1.65)	42.5	1.12 (0.77-1.62)	41.4	0.73 (0.51-1.05)
Co-Morbidities	No (n=392)	43.6	1.00	58.7	1.00	18.6	1.00	41.6	1.00	44.9	1.00
	Yes (n=89)	37.1	0.76 (0.47-1.22)	55.1	0.86 (0.54-1.37)	19.1	1.03 (0.57-1.85)	40.4	0.95 (0.60-1.52)	43.8	0.96 (0.60-1.52)
Knew someone with cancer	No (n=400)	42.0	1.00	58.5	1.00	19.5	1.00	42.8	1.00	44.3	1.00
	Yes (n=81)	44.4	1.10 (0.68-1.79)	55.6	0.89 (0.55-1.43)	14.8	0.72 (0.37-1.39)	34.6	0.71 (0.43-1.17)	46.9	1.11 (0.69-1.80)
Variables		Too busy to make time to go to the doctor		Too many other things to worry about		Difficult for me to arrange transport to the doctor’s surgery		Worried about what the doctor might find		Not feeling confident talking about my symptom with the doctor	
		%	OR	%	OR	%	OR	%	OR	%	OR
			(95% CI)		(95% CI)		(95% CI)		(95% CI)		(95% CI)
Gender	Male (n=258)	61.6	1.00	51.2	1.00	39.9	1.00	61.6	1.00	40.3	1.00
	Female (n=223)	57.8	0.85 (0.59-1.23)	45.3	0.79 (0.55-1.13)	44.4	1.20 (0.84-1.73)	73.1	1.69 (1.15-2.49)	45.7	2.49 (0.46-1.25)
Age	15-16 (n=201)	58.7	1.00	45.8	1.00	36.3	1.00	69.7	1.00	41.3	1.00
	17 (n=280)	60.7	1.09 (0.75-1.57)	50.4	1.20 (0.84-1.73)	46.1	1.50 (1.03-2.17)	65.0	0.81 (0.55-1.19)	43.9	1.19 (0.44-1.11)
Co-Morbidities	No (n=392)	62.0	1.00	50.3	1.00	43.4	1.00	67.6	1.00	43.9	1.00
	Yes (n=89)	50.6	0.63 (0.39-1.00)	40.4	0.67 (0.42-1.07)	36.0	0.73 (0.46-1.18)	64.0	0.85 (0.53-1.38)	38.2	1.38 (0.38-0.79)
Knew someone with cancer	No (n=400)	59.3	1.00	50.0	1.00	42.5	1.00	65.5	1.00	42.8	1.00
	Yes (n=81)	63.0	1.17 (0.71-1.91)	40.7	0.69 (0.42-1.12)	39.5	0.88 (0.54-1.44)	74.1	1.50 (0.88-2.58)	43.2	2.58 (0.43-1.02)

## Discussion

To the best of our knowledge this is the first study conducted in a Middle-East Arabic country to identify adolescent awareness levels of cancer risk factors, the warning signs, the anticipated time to seeking medical help and the perceived barriers to seeking medical help. The current study indicated that the recognition levels of cancer risk factors and symptoms among adolescents in Oman is low (the average awareness of risk factors was 36% and 39.6% for symptoms). A similar study from the UK which used the CAM questionnaire showed that awareness levels among adolescents of cancer symptoms was also low, a similar finding to the current study (Kyle et al., 2013). Previous studies conducted in Oman among adults indicated low awareness levels in identifying risk factors and symptoms for the most common cancers, and the levels were also low for specific types of cancer, such as colorectal and ovarian (Al-Azri et al., 2015; Al-Azri et al., 2016; Al-Azri et al., 2014).

Learning experiences in adolescents (experiences, exploration, reflection, emotional learning, developing teamwork skills, forming ties with community members) have been shown to affect their experiences in adulthood (Hansen et al., 2003). Educational authorities in the USA have recognised the importance of integrating the subject of oncology in schools’ curriculums, leading the students to be exposed to many different types and causes of cancer and informing them of modern treatment options that address individual patient needs (Dieuwertje etal., 2018). It has been found that teaching adolescent males (age 15–20 years) at high school and college about testicular cancer significantly increases their knowledge and awareness of risk factors, cancer symptoms and how to carry out testicular self-examination in adulthood (Klein et al., 1990). Healthcare authorities should liaise with educational authorities in order to integrate fundamental cancer information into school curriculums, to improve adolescent cancer awareness levels as part of early cancer prevention (Klein et al., 1990). The World Health Organisation has reported on a well-established school health programme in Oman and has recognized the close collaboration between the Ministries of Health and Education in delivering multiple interventions to promote the students’ health in schools (World Health Organization, 2014).

The current study found that there were low recognition levels of cancer lifestyle risk factors among the adolescents (being overweight, doing less physical exercise, an unhealthy diet), as well as the infection-related risk factors (HPV, hepatitis B), which is very alarming. Previous studies conducted in Oman have shown a high prevalence in obesity and a low tendency to partake in physical activities among adolescents, and a rise in both of these factors has been associated with an increased cancer incidence (Al-Kilani et al., 2012; Bray et al., 2018). This study showed that adolescents who reported having existing co-morbidities were less aware that eating fewer fruits and vegetables attributed the risk of developing cancer. 

Although traditional Arabic countries, including Oman, have a lower rate of HPV infections due to more conservative sexual behaviours, the prevalence of HPV infections is increasing at an alarming rate as a result of increased levels of sexual activity among the younger population (10-24 years old) (Baghi et al., 2017). Notably, the majority of women in these countries were not aware of the availability of the HPV vaccination, and/or did not accept the vaccine (Ortashi et al., 2013; Ortashi et al., 2014). Fear of side effects, absence of clear benefits and objections from religious authorities have been listed as reasons for not being accepting of the HPV vaccine (Ortashi et al., 2013). 

The higher recognition levels among the adolescents in this study for smoking and drinking alcohol as risk factors of cancer compared to other studies might be due to the conservative nature of Omani society. The majority of people in Oman are Muslims, and alcohol intake is forbidden as part of their religious faith. Despite the fact that smoking in Oman is not common (prevalence of smoking among adults is 4.6%) in comparison to other Arabic countries, the government has been successful in warning the public about the dangers of tobacco, and is even enforcing a law to ban smoking in public places throughout the country (Al-Lawati et al., 2017). 

There are numerous activities conducted in schools and colleges, including mass media, to augment awareness of the harms of tobacco. Thus, negative perceptions are possibly reflected in the societal attitudes and mass media efforts, rather than schooling, since 83.9% of the participants in a previous study reported seeing anti-tobacco media messages, but only two-thirds of young people aged 13–15 years reported that they were taught of the dangers of smoking (Al-Lawati et al., 2017). Smoking and alcohol are regarded by many Muslims to be “social stigma” and people tend not to drink alcohol or smoke in order to protect their health and faith (Ahmed, 2013).

The higher levels of knowledge of cancer risk factors, signs and symptoms among girls compared to boys in this study might be related to the fact that girls usually excel in comparison to boys with regards to knowledge of medicine, though boys had higher knowledge levels in other fields (Tran et al., 2014). In the Muslim culture, girls tend to spend more time on homework compared to boys, and are given less freedom to spend time outside of the home, as parents often felt anxious of their liaising with boys, which would bring perceived shame (Amanda, 2017) They also have fewer choices for university and jobs in comparison to boys, and they must study hard to get high scores in school exams to be admitted to universities and work in reputable jobs such as teachers or doctors (Tran et al., 2014). 

Adolescents who reported to know someone with cancer were significantly more aware of some of the cancer risk factors, as expected. Close family members who care for cancer patients would have the chance to receive information from healthcare providers, aid in treating and caring for the patient, and be involved in their treatment decision making, which would increase their cancer knowledge levels (Al-Bahri et al., 2018). Also, being exposed to patients who have been diagnosed with cancer has been associated with greater levels of health-related knowledge, information and meeting healthcare professionals, which increases cancer awareness levels and knowledge (Al-Bahri et al., 2018). This means that adolescents who have experienced knowledge of cancer are more likely to understand the cancer symptoms.

Female adolescents in this study were more likely than the males to identify several emotional barriers from seeking medical help, such as “embarrassment”, “being scared” and “worried about what the doctor might find”. There is a paucity in the literature regarding teenage cancer diagnosis delay; one study from the UK found that the reasons for delay were mainly attributable to the actions of healthcare professionals (Fern et al., 2011) Previous studies conducted in Oman and in the UK showed that women were more likely than men to perceive similar emotional barriers to the ones listed above which would prevent them from seeking early medical help (Al-Azri et al., 2016; Robb et al., 2009). Women have been noted to worry about cancer symptoms, due to being worried about side effects of treatments or because they believed that there was no cure for cancer, which could lead to a delay in their diagnosis (Murphy et al., 2018). Fear of cancer diagnosis, embarrassment and fear that their family might think the symptoms are psychosomatic, were found to be the most common contributing factors delaying seeking medical help (Smith et al., 2005).

In Oman, being a Muslim women and having a higher level of modesty and self-rated religiousness, or living in a conservative culture, might attribute to the delays in seeking medical help, as they might feel of embarrassed to talk to or be examined a male doctor (Rizk et al., 2005; Vu et al., 2016). Despite the fact that there are Omani and expatriate female physicians available, women who think that it might be difficult to see a doctor anticipated a waiting time in seeking medical help for cancer warning signs or symptoms which was significantly longer than the women who did not have these thoughts (Quaife et al., 2014). This study has suggested that interventions to improve early cancer diagnosis among adolescents (particularly female) should focus on addressing the emotional barriers. However, more research on why there are delays in cancer diagnosis and intervention among adolescent females are required, to understand the interplay of psychosocial development and changes in cancer awareness levels.

Although the majority of adolescents in this study struggled to identify the signs and symptoms of cancer, the majority (88.8%) reported that they would seek medical help within the first two weeks, particularly for obvious symptoms such as bleeding, swelling, and pain (in comparison to vague symptoms such as loss of appetite or weight). Previous studies conducted among adolescents in the UK and also among Omani adults have reported similar findings (Al-Azri et al., 2015; Kyle et al., 2012). Sufficient knowledge levels of the signs and symptoms of cancer are a prerequisite for the correct interpretation and seeking medical help (Macleod et al., 2009). 

Obvious cancer symptoms, such as swelling, symptoms that cause pain or interfere with daily function, usually trigger an individual to seek medical help earlier than if the symptoms are vague, such as tiredness or weight loss (Robb et al., 2009). It has been found that patients with breast cancer were less likely to delay in seeking help and patients with prostate and rectal cancer were most likely to delay (Forbes et al., 2014). Conversely, it can be argued that seeking medical help at an early stage does not always indicate that the patients are aware of the signs and symptoms of cancer, as this knowledge might not be entirely predictive of help seeking behavior (Sheikh and Ogden, 1998). 

There are several limitations in this study. Firstly, the current study was conducted among Omani adolescents in one area of Muscat, the capital city of Oman, which could affect the generalizability. However, we believe that this is not a major issue, as most people living in Muscat are originally from other regions of Oman. In the future, a larger, national study is needed for better representative sampling. Another limitation is that, although we translated the adult CAM questionnaire to Arabic, there might have been some disparity in the meaning of some of the translated statements, which was beyond our control. It should be noted though that the Cronbach’s alpha of the Arabic version of the ovarian CAM from the pilot study was found to be high. This study also examined adolescents’ perceptions of barriers to seeking help, which may not be the same as actual help-seeking behavior. Lastly, although the adolescents who had a family history of cancer might create some interference or bias, the CAM questionnaire did not recommend excluding the participants with family history. 

In conclusion, this study showed for the first time that the majority of adolescents in Oman have low awareness levels of the cancer risk factors and symptoms. The Ministry of Education in Oman has considered further steps to integrate fundamental cancer knowledge in the school teaching curriculum to increase cancer awareness levels. Teaching programs should aim to promote health education by teaching cancer prevention, health risks and desirable lifestyles, such as healthy eating and exercise. Continuing to promote cancer education through school health programs might enhance their effectiveness. Also, involvement of healthcare professionals, such as general practitioners and community nurses, as visiting guest speakers in the program should be considered. 

Emotional barriers were as the most commonly reported barrier to seeking medical help, particularly among female adolescents. The targeting Omani female adolescents in schools to promote positive health-related behaviours is needed to eliminate emotional barriers, such as being scared, embarrassment, being worried and fear of cancer. Educating adolescents of the importance of building trust at an interpersonal level with their healthcare professionals and disclosing possible cancer symptoms should be emphasised. Educate female adolescents and reassuring them of the availability of female doctors in their local health centres and hospitals might decrease some of the emotional barriers and improve early cancer detection levels. Education programs should highlight to female adolescents of the availability and the importance of attending cancer screening, such as Pap smears for cervical cancer and mammographies for breast cancer. The availability of the HPV vaccine should also be highlighted, though this might prove to be difficult to be accepted in conservative Islamic societies. 

## Compliance with Ethical Standards

This study has been approved by the Medical Research and Ethics Committee of the College of Medicine and Health Sciences at Sultan Qaboos University, Muscat, Oman (MREC # 1360). Written consent forms were obtained from the parents, guardians and also from each student before completion of the CAM questionnaire.

## Conflict of Interest Statement

The authors declare that they have no conflict of interest.

## Funding

None.
